# *Allium sulaimanicum*: A new *Allium* species and section from Pakistan

**DOI:** 10.3389/fpls.2022.1020440

**Published:** 2022-12-14

**Authors:** Nazar Khan, Nikolai Friesen, Amir Sultan, Reinhard M. Fritsch, Tahir Khan, Kamran Ishaq

**Affiliations:** ^1^ Department of Botany, Government Degree College, Zhob, Balochistan, Pakistan; ^2^ Botanical Garden of Osnabrück University, Osnabrück, Germany; ^3^ National Herbarium, National Agricultural Research Centre, Islamabad, Pakistan; ^4^ Leibniz Institute of Plant Genetics and Crop Plant Research, Gatersleben, Germany; ^5^ Department of Botani, Goverment Girls Degree College, Zhob, Balochistan, Pakistan; ^6^ Department of Agricultural Extension, Zhob, Balochistan, Pakistan

**Keywords:** Sulaiman range, ITS, *rpl*32-*trn*L, phylogeny, chromosome

## Abstract

A new species, *Allium sulaimanicum*, is described from northern Balochistan and southern Khyber Pakhtunkhwa in Pakistan based on morphological, molecular, and cytological studies. The new species is characterised by long runner-like cylindrical rhizomes of adult plants, cylindrical bulbs, linear leaves with minute soft hairs along veins, campanulate perigonium, and white to creamy white, ovate to elliptical, 4.5–5-mm-long acute tepals, with brownish to purplish nerves, stamens as long as to slightly longer than tepals, yellow to brick red anthers, hexagonal ovary, and white and papillate/warty along angles. The presence of long herbaceous rhizomes indicated serious isolation of the new species; hence, a new section *Sulaimanicum* is proposed to accommodate the new species. The new species is diploid with a chromosome number of 2n = 16. Detailed morphological description, illustrations, phylogenetic analyses based on sequences of plastid spacers (*rpl*32-*trn*L (UAG) and *trn*Q-*rps*16) and nuclear ITS, karyotype features, and a distribution map of the new species are provided.

## Introduction

*Allium* L. (Amaryllidaceae J.St.-Hil.: Allioideae Herb.) is one of the largest monocot genera with currently more than 1,000 accepted species (Govaerts et al., 2021) mainly distributed throughout the Northern Hemisphere (Stearn, 1978; Stearn, 1992; Fritsch & Friesen, 2002; Friesen, 2022). The key centres of biodiversity are located in arid and sub-arid regions of Southwestern and Central Asia, occupying areas of the Mediterranean extending to the west of Asia Minor. Another considerably smaller centre is in western North America (Friesen et al., 2006; Nguyen et al., 2008; Li et al., 2010; Wheeler et al., 2013; Friesen, 2022). The genus is characterised by bulbs (often formed on rhizomes) enclosed in membranous, fibrous, or reticulate tunics, free or basally connate sepals, and usually a subgynobasic style (Friesen et al., 2006). The genus *Allium* is a member of the family Amaryllidaceae, subfamily Allioideae, and tribe Allieae (Chase et al., 2016; Friesen, 2022). Over 50 *Allium* species are widely grown globally or locally as cultivated plants. Also, very many wild species are collected by local people in nature as vegetables, for medicinal purposes, or as ornamental plants (Fritsch & Abbasi, 2013).

The enormous morphological, cytological, and genetic variabilities are reflected in the complicated taxonomic structure of the genus, consisting of 15 subgenera and 72 sections (Friesen et al., 2006), and more than 10 new sections were described during the last 10 years. All subsequent phylogenetic studies on the genus *Allium* have confirmed the three evolutionary lineages in the genus, including the monophyletic origin of all subgenera in the first and second evolutionary lineages (Li et al., 2010; Wheeler et al., 2013; Hauenschild et al., 2017; Xie et al., 2019; Costa et al., 2020; Xie et al., 2020; Friesen, 2022). The phylogenetic relationships in the third (youngest) lineage are less clear. According to the latest studies, many subgenera in the third lineage are not monophyletic. This mainly affects the subgenera *Allium*, *Cepa* (Mill.) Radić, *Reticulatobulbosa* (Kamelin) N. Friesen, *Rhizirideum* (G. Don ex Koch) Wendelbo, and *Polyprason* Radić (Li et al., 2016; Hauenschild et al., 2017; Xie et al., 2019; Friesen et al., 2020a; Xie et al., 2020; Friesen, 2022).

Forty-one *Allium* species were recorded in Flora of Pakistan (Nasir, 1975), with the description of *Allium zhobicum* N. Khan, A. Sultan & R. M. Fritsch and the addition of *Allium caroli-henrici* Wendelbo and *Allium registanicum* Wendelbo as new records for Pakistan (Khan et al., 2021); the genus representation in Pakistan was extended to 44 species.

During exploratory fieldwork in the Shinghar area (Sulaiman range) of Sherani district and Tor Ghar area of Musakhail District in northern Balochistan and the Sulaiman range in Dera Ismail Khan District of Khyber Pakhtunkhwa in 2019–2021 by Nazar Khan, Tahir Khan, and Kamran Ishaq, some *Allium* species were collected. Photos of two *Allium* species were sent to Reinhard Fritsch and Nikolai Friesen for identification. One of these was identified as *Allium roylei* Stearn, while the other plant, collected in the Sulaiman range, turned out to be an unfamiliar species. According to the morphological characters (inflorescences, flowers, and leaves), it was supposedly a new species from the *Dagestanica* section in the genus *Allium*, but the long herbaceous rhizome indicated serious isolation of this species. Leaf and seed samples were subsequently sent to Germany (Osnabrück) for molecular and cytological analyses. Molecular and cytological analyses have clearly shown the peculiarity of this species. In this work, we describe this plant as a new species, *Allium sulaimanicum*, and analyse its relationship and position in the *Allium* classification. With the addition of *A. sulaimanicum*, this genus is now represented by 45 species in Pakistan.

## Material and methods

### Taxon sampling

Plants of four accessions of *A. sulaimanicum* were collected in the Sulaiman Mountains during 2019–2021. DNA was isolated from silica gel-dried leaves. Newly sequenced accessions are marked with Am number in the trees, and their origin is shown in **Table 1**. To determine the position of the *A. sulaimanicum* in the genus, nuclear ITS sequences and *rpl*32-*trn*L (UAG) plastid fragments of representative species from most sections of the third evolutionary line were taken from NCBI GenBank (https://www.ncbi.nlm.nih.gov/nucleotide/). Some accessions from the first and second evolutionary lines were selected as outgroups (Friesen et al., 2006). Sequences from NCBI GenBank are marked with GenBank accession numbers on the trees. To find the closest relatives for *A. sulaimanicum*, we checked ITS, *rpl*32-*trn*L (UAG), and *trn*Q-*rps*16 sequences using the Nucleotide BLAST program of the NCBI GenBank (https://blast.ncbi.nlm.nih.gov/Blast.cgi).

**Table 1:** Origin, source, and GenBank accession numbers of *Allium sulaimanicum* used for phylogenetic analyses.

Accession	Species	Coordinates	Country	Voucher	*nr*ITS	*trn*Q-*rps*16	*rpl*32-*trn*L
Am1153	*A. sulaimanicum*	31.552778N, 69.9175 E	PAK	RAW 101507	OP218571	OP210292	OP210288
Am1154	*A. sulaimanicum*	31.603333N, 69.966111E	PAK	RAW 102202	OP218572	OP210293	OP210289
Am1300	*A. sulaimanicum*	31.6075 N, 69.732778 E	PAK	RAW 102200	OP218573	OP210294	OP210290
Am1301	*A. sulaimanicum*	31.295833 N, 70.023611 E	PAK	RAW 102201	OP218574	OP210295	OP210291

### DNA extraction, amplification, and sequencing

Total genomic DNA was isolated from leaves, dried in silica gel using the InnuPREPP Plant DNA Kit (Analytik Jena AG, Jena, Germany) according to the manufacturer’s instructions, and used directly in PCR amplification. The complete nuclear ribosomal ITS region (ITS1, 5.8S, and ITS2) was amplified using the primers ITS-A (Blattner, 1999) and ITS-4 (White et al., 1990). The PCR conditions for ITS followed those of Friesen et al. (2006). PCR conditions and primers for the chloroplast regions *trn*L-*rpl*32 and *trn*Q-*rps*16 were described in Shaw et al. (2007). PCR products were sent to Microsynth SeqLab (Göttingen, Germany; www.microsynth.seqlab.de) for sequencing. The sequences from all the individuals were manually edited in Chromas Lite 2.1 (Technelysium Pty Ltd., South Brisbane, QLD, Australia) and aligned with ClustalX (Thompson et al., 1997), and the alignment was manually corrected using MEGA 7 (Kumar et al., 2016).

### Phylogenetic analyses

Both data sets (nrITS and the cpDNA *trn*L-*rpl*32 markers) were analysed separately for position identification in the third evolutionary lineage and to find the closest relatives of *A. sulaimanicum* through Fitch parsimony with the heuristic search option in PAUP version 4.0b10 (Swofford, 2002) with MULTREES, TBR branch swapping, and 100 replicates of random addition sequence. Gaps were treated as missing data. The consistency index (CI) (Kluge & Farris, 1969) was calculated to estimate the amount of homoplasy in the character set. The most parsimonious trees returned by the analysis were summarised in one consensus tree using the strict consensus method. Bootstrap analyses (bootstrap support (BS)) using 1,000 pseudoreplicates were performed to assess the support of the clades (Felsenstein, 1985). Bayesian phylogenetic analyses were also performed using MrBayes 3.1.23 (Ronquist & Huelsenbeck, 2003). The sequence evolution model was chosen following the Akaike information criterion (AIC) obtained from jModelTest2 (Dariba et al., 2012). Two independent analyses with four Markov chains were run for 10 million generations, sampling trees every 100 generations. The first 25% of trees were discarded as burn-in. The remaining 150,000 trees were combined into a single data set, and a majority-rule consensus tree was obtained along with posterior probabilities (PP). The alignments of both data sets can be found in Dryas: https://doi.org/10.5061/dryad.73n5tb313.

### Cytology

Seeds were germinated in petri dishes on damp filter paper. The germinated seeds with root tips longer than 10 mm were kept on ice in distilled water overnight. They were then transferred to room temperature for 20 min and pre-treated for 3 h at room temperature in an aqueous solution of 0.1% colchicine. Roots were then fixed in a freshly prepared mixture of 96% ethanol and glacial acetic acid (3:1 v/v). Root tips were stained using hematoxylin according to the protocol reported by Smirnov (1968). Well-spread metaphase plates were electronically documented (digitally photographed), and finally, the chromosomes of the best plates were measured and pairwise arranged using the KaryoType software (Altinordu et al., 2016). Because the idiograms automatically assembled by the software were not satisfactory, we manually ordered the chromosome pairs according to their length and shape. The idiograms were designed using the bar graph function implemented in MS Excel^®^. The terminology of Levan et al. (1964) was applied. The karyotype was constructed using seven metaphase plates.

## Results

### Morphological description and distribution

**Figure 1:**
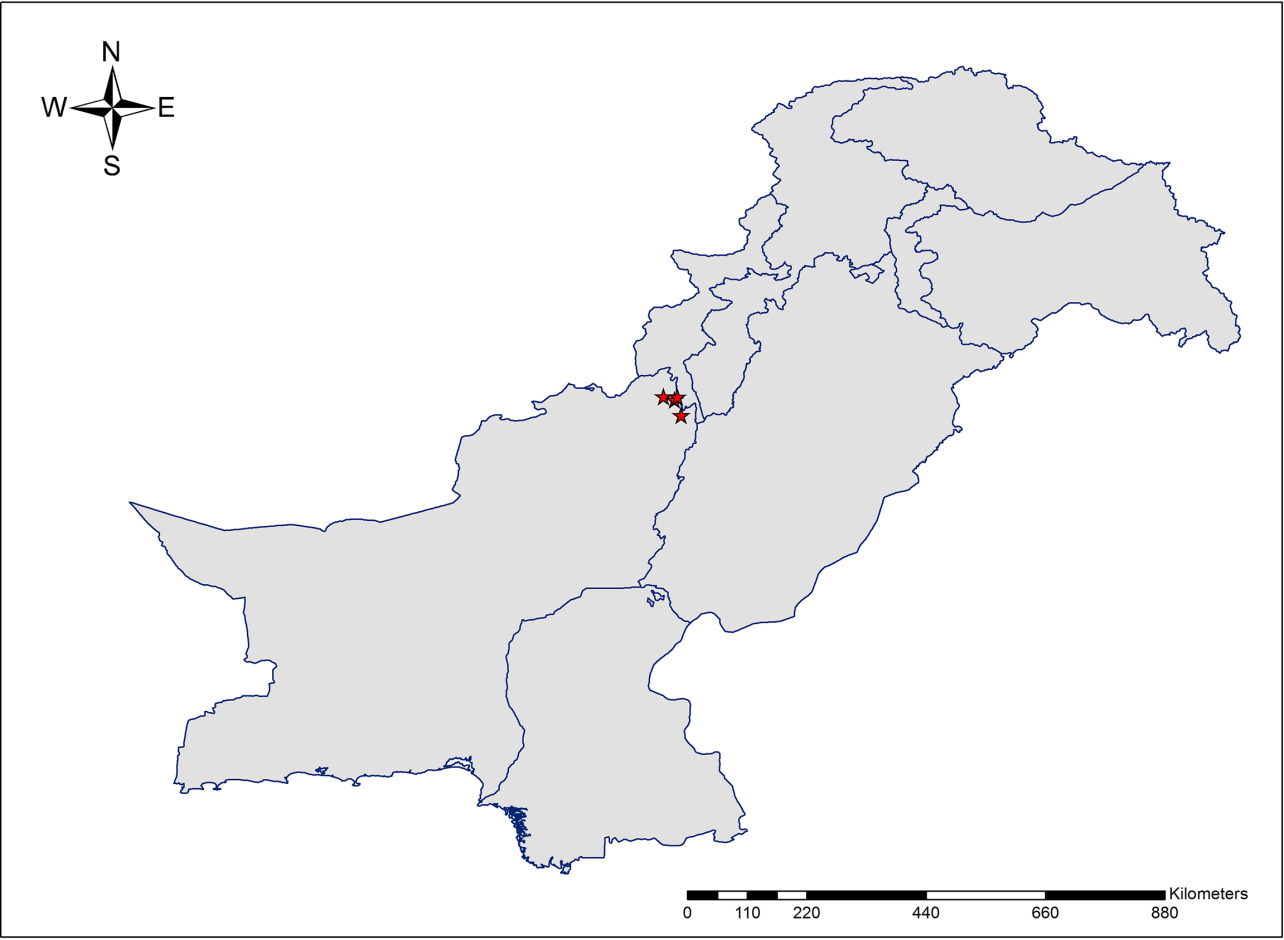
The geographic location of collected accessions of *Allium sulaimanicum* in Pakistan.

**Figure 2:**
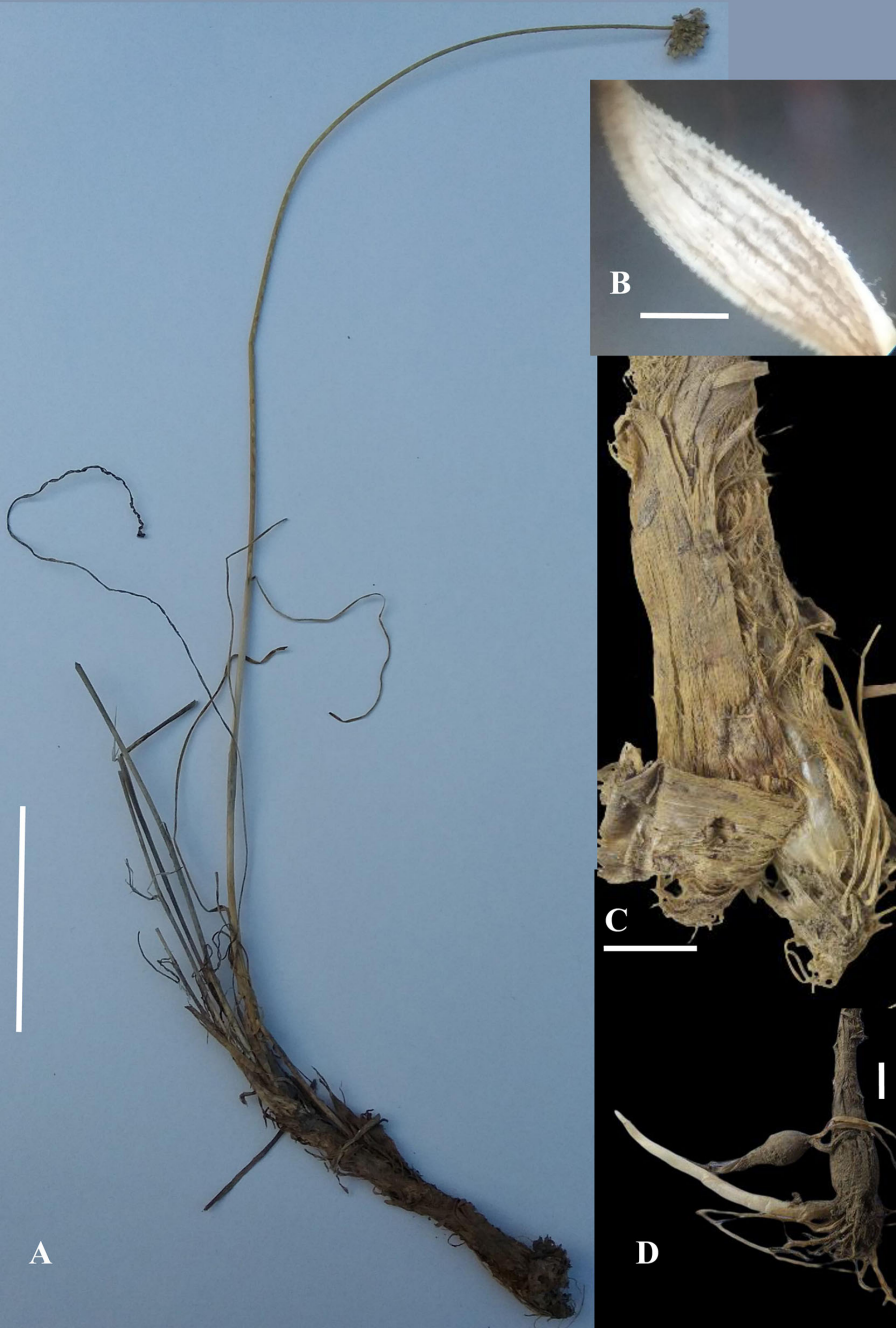
*Allium sulaimanicum*: **(A)** whole plant (scale bar: 8 cm) **(B)** Leaf in enlargement under a stereomicroscope (scale bar: c. 1 mm) **(C)** Bulb with outer coats brown coriaceous at the extreme base and otherwise fibrous with strips (scale bar: 6 mm) **(D)** Bulb with long rhizome (scale bar: 6 mm) (Photos by Nazar Khan except Fig. 2A by Amir Sultan and Fig. 2C by Muhammad Saleem).

*A. sulaimanicum* N. Khan, A. Sultan et N. Friesen sp. nov. **Figures 1** and **2**.

Holotype: Khyber Pakhtunkhwa, Dera Ismail Khan District, Kaisa Ghar, Ahmadi Dirga, 31°33'10" N, 69°55'3" E, 2,022 m, N. K. *Mandokhel*, *K. I. Bahadikhel & T. Khan*, 16 July 2020 (RAW 101507).

### Description

Bulb cylindrical, 4–9.5 cm long, 4–10 mm broad at the base, outer coats brown coriaceous at the extreme base and otherwise fibrous with strips, inner coats white membranous, in adult plants on long cylindrical rhizomes. Scapes 1–2, usually 1 with persistent old scapes, 25–45–(50) cm tall, covered at the base with scabrid leaf sheaths, cylindrical, bearing ridges and furrows, scabrid. Leaves linear 2–3, shorter than scape, 8–25 cm long, 1–3 mm broad, papillate along margins and veins, glabrous to sparsely pilose along veins with white hairs turning brown upon drying, veins 3–9. Spathe white with brownish veins and acuminate tips, split longitudinally unequally into two lobes. Umbel hemispherical, lax to densely flowered, pedicels unequal, 8–15 mm long, white to greenish at tips. Perigonium campanulate, tepals white to creamy white, 4.5–5 mm long, nerves brownish to purplish, ovate to elliptical, acute, recurved, inner tepals slightly broader at the base than outer. Stamens as long as to slightly longer than tepals, filaments connate at base and adnate to tepals, triangular at base, inner filaments slightly more dilated than outer at the base. Anthers yellow to brick red, 1–1.2 mm, basifixed. Ovary hexagonal, white, papillate/warty along angles, style 2–5 mm, stigma simple to slightly capitate sometime exserted. Seeds black, 1.5–2.5 × 0.8–1.2 mm, oblong to semi-orbicular, testa cells ± elliptical, periclinal walls ± even with 1–3 verrucae (**Figure 2D**).

#### Diagnosis

From the presumably related *Allium maowenense* J. M. Xu (NC Sichuan, China), it differs with open star-shaped flowers (closed in *A. maowenense*), stamens equal or only slightly longer than tepals (twice longer in *A. maowenense*), and cylindrical bulb (not ovoid). From *Allium matiniae* N. Friesen & M. Abbasi (NW Iran), it differs with stamens equal or only slightly longer than tepals (much shorter in *A. matiniae*) and base of inner filaments slightly more dilated than the outer ones (equal in *A. matiniae*). *A. sulaimanicum* differs from both species with long runner-like rhizomes.

#### Vernacular name

Da Khara Khokhai

#### Etymology

The epithet refers to the Sulaiman mountain range.

#### Distribution and conservation status

**Figure 3:**
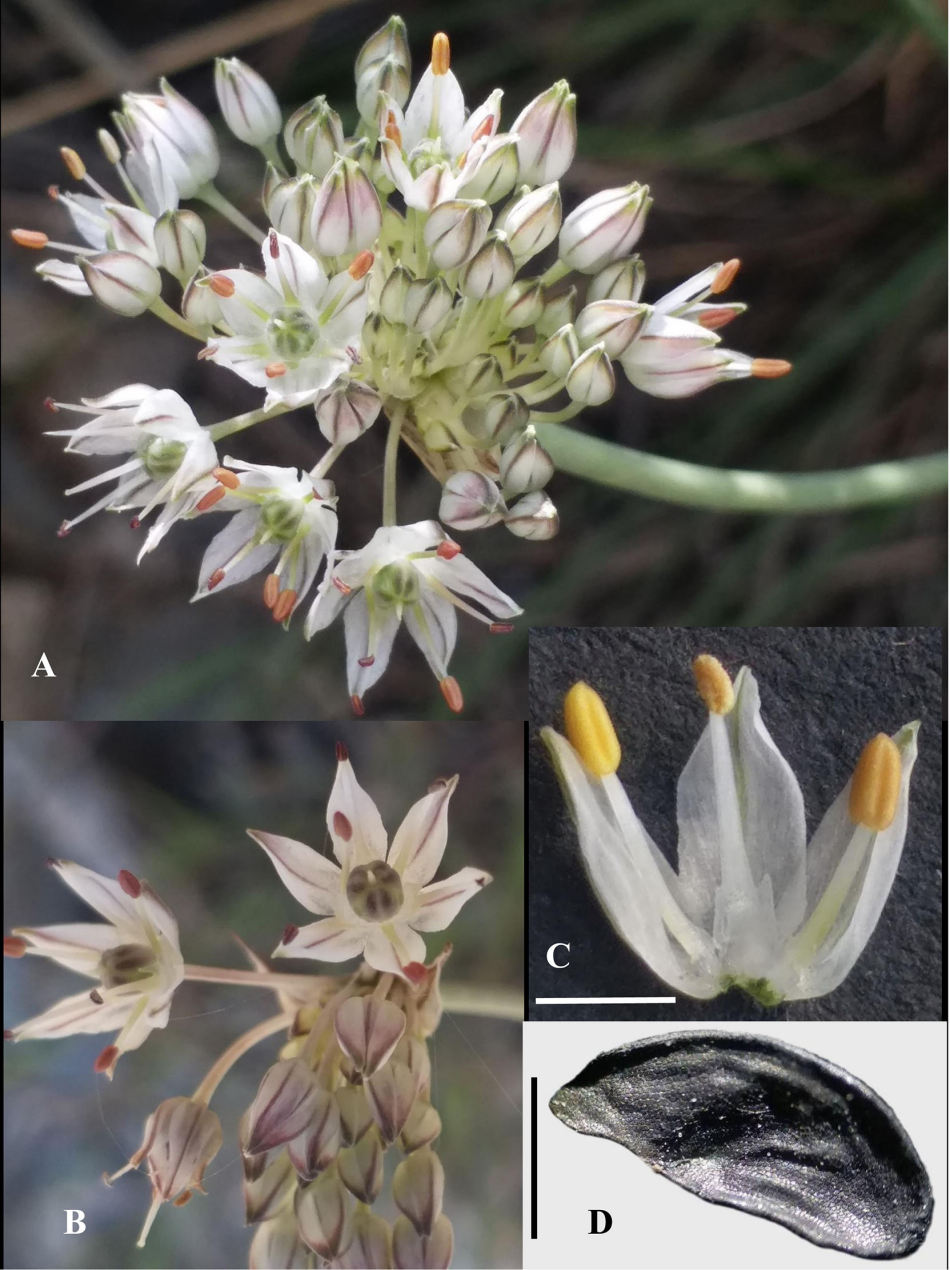
*Allium sulaimanicum*. **(A, B)** Inflorescence **(C)** Flower section with tepals and stamens (scale bar: c 1.5 mm) **(D)** seed (scale bar: c 1 mm) (Photos by Nazar Khan).

The new species is endemic to Pakistan and currently known from the Sulaiman range in southern parts of Dera Ismael Khan District and northern districts (Sherani and Musakhail) of Balochistan (**Figure 3**). Based on the current known distribution of *A. sulaimanicum*, its extent of occurrence is determined as 377.268 km^2^. However, further exploration in similar habitats in the Sulaiman range may yield further populations. Given the fire incidents in the *Pinus gerardiana* Wall. ex D.Don forest, it requires conservation measures.

### Other specimens examined

Pakistan, Khyber Pakhtunkhwa, Dera Ismail Khan District, Takht-e-Sulaiman, 31 36 12 N, 69 57 58 E, 3000 m, *N. K. Mandokhel*, *K. I. Bahadikhel & T. Khan*, 16 July 2020 (RAW 102202); Pakistan, Balochistan, Sherani District, Shinghar, 31 36 27 N, 69 43 58 E, 2,800 m, *N. K. Mandokhel & T. Khan*, 15 September 2019 (RAW 102200); Musakhail District, Tor Ghar (Mizri Ghar), 31 17 45 N, 70 01 25 E, 2,366 m, *N. K. Mandokhel*, *K. I. Bahadikhel & T. Khan*, 19 September 2021 (RAW 102201).

### Habitat and ecology

Found in shady places under chilgoza pine trees (*P. gerardiana*) or other shrubs and among herbaceous plants/grasses growing in gravelly loam to humus rich brownish to blackish soil at elevations of c. 2,000–3,000 m a.s.l.

### Ethnobotany

Plants are not used by local people; flowers are grazed by goats.

### Conservation status

The extent of occurrence: 377.268 km^2^

*Allium* subgen. *Polyprasum* Radić, *Allium* sect. *Sulaimanicum* N. Friesen sect. nov.

Typus: *A. sulaimanicum* N. Khan, A. Sultan et N. Friesen

Long runner-like cylindrical rhizomes of adult plants bear long-cylindrical bulbs with coriaceous tunics dissolving in strips and finally in reticulate fibres. Leaves linear. Perigonium campanulate, tepals white to creamy white, nerves brownish to purplish.

### Phylogenetic analyses

Sequences of all four accessions of *A. sulaimanicum* (Am1153, Am1154, Am130, and Am1301) are identical in all sequenced plastid spacers (*rpl*32-*trn*L (UAG), *trn*Q-*rps*16, and *trn*L-*trn*F) and nuclear ITS.

#### Blast analysis

##### ITS

Blast analysis in the NCBI GenBank with ITS sequence of *A. sulaimanicum* accession Am1154 shows 86%–87% similarity with *Allium* species of *Allium* subgen. *polyprason*: HG794233 *Allium* cf. *talassicum*, 87.33%; FM945429 *Allium hymenorhizum*, 86.79%; HG794231 *Allium kasteki*, 86.79%; OM030262, OM030260 *Allium palentinum*, 86.64%; AJ250290 *Allium carolinianum*, 86.66%; HG794166 *Allium tianschanicum*, 87.18%; MF675014–MF675023 *Allium forrestii*, 86.57%.

##### *rpl*32*-trn*L (UAG)

The 10 best hits with *rpl*32-*trn*L spacer in the Blast analysis of NCBI GenBank are representatives of the *Allium* sect. *Daghestanica*: LR700256 *A. matiniae*, 95.41%; LR700253, LR700254 *Allium daghestanicum*, 95.40%; LR700250, LR700251, LR700252 *Allium gunibicum*, 95.40%; LR700255 *A. daghestanicum*, 95.28%; NC_042154 *Allium chrysanthum*, 93.76%; MH383268 *Allium chrysocephalum*, 93.65%; MH383267 *Allium rude*, 93.54%, followed in between with MK820610 *Allium caeruleum*, 93.34%, from *Allium* sect. *Coerulea*.

##### *trn*Q-*rps*16

Blast analysis in the NCBI GenBank with sequences of *trn*Q-*rps*16 of *A. sulaimanicum* shows very strong similarity with representatives of the *Allium* subgen. *Cepa*: MH159130 *Allium altaicum* and LT674586 *Allium fistulosum*, 98.51%; MT160180, NC_050981, MT300497, MT300496 *Allium galanthum*, 98.16%; KM088014, KF728079, *Allium cepa* 98.05%; MZ019479 *Allium semenovii*, 93.93% and LT699700, NC_057575, MN519208 *Allium schoenoprasum*, 97.48%.

### Position of *Allium sulaimanicum* in the third evolutionary lineage

#### ITS analysis

**Figure 4:**
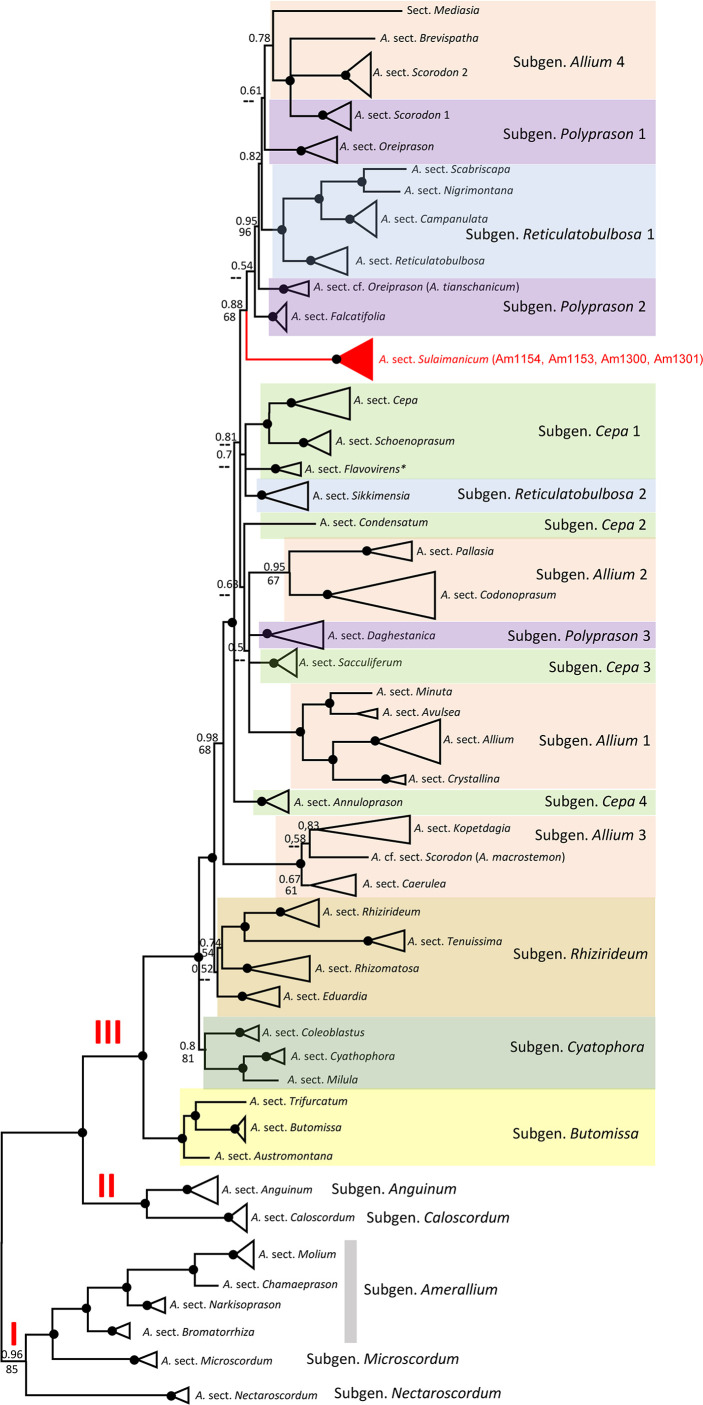
Generalised nrITS tree of the third evolutionary line of genus *Allium*. Numbers by nodes represent bootstrap support (1,000 replicates) and Bayesian probabilities. Roman numerals (I, II, and III) designate clades of three evolutionary lines. The joint presence of Bayesian probabilities over 0.98 and bootstrap support over 95% is indicated with a black dot.

The alignments of nrITS sequences (including the 5.8S gene) with 232 accessions of *Allium* species, comprising a selection of representatives from each subgenus and sections of the third evolutionary line, including four accessions of *A. sulaimanicum*, consist of 727 characters of which 510 variable characters are parsimony informative. Unweighted parsimony analysis of the 232 sequences resulted in about 14 million most parsimonious trees of 4,501 steps (CI = 0.2543). The substitution model TVM+G was chosen by AIC in JModeltest-2.1.7 for the Bayesian analysis. The parsimony and Bayesian analyses produce identical topology (see **Figure S1**). All *A. sulaimanicum* accessions form a monophyletic clade and stand alone in the third evolutionary line between representatives of the subgenera *Polyprasum* and *Cepa* (see **Figure S1**). The generalised ITS tree with sections and subgenera names is shown in **Figure 4**. Some subgenera in the third evolutionary lineage according to Friesen et al. (2006) are not monophyletic: this applies to *A.* subgenera *Cepa*, *Reticulatobulbosa*, *Polyprason*, and *Allium*. *A. sulaimanicum* accessions form a separate monophyletic clade inside the third evolutionary lineage: Bayesian PP = 1 and BS = 100 present strong support in the phylogenetic analyses.

#### CP analysis

The alignments of *rpl*32-*trn*L (UAG) sequences with 99 accessions of diverse *Allium* species (a selection of representatives from each subgenus and sections of the third evolutionary line, including four accessions of *A. sulaimanicum*) consist of 1,046 characters of which 282 variable characters are parsimony informative. Unweighted parsimony analysis of the 99 sequences resulted in about 1.541.841 most parsimonious trees of 841 steps (CI = 0.6385). The substitution model GTR+G was chosen by AIC in JModeltest-2.1.7 for the Bayesian analysis. The parsimony and Bayesian analyses produced identical topologies (see **Figure S2**).

**Figure 5:**
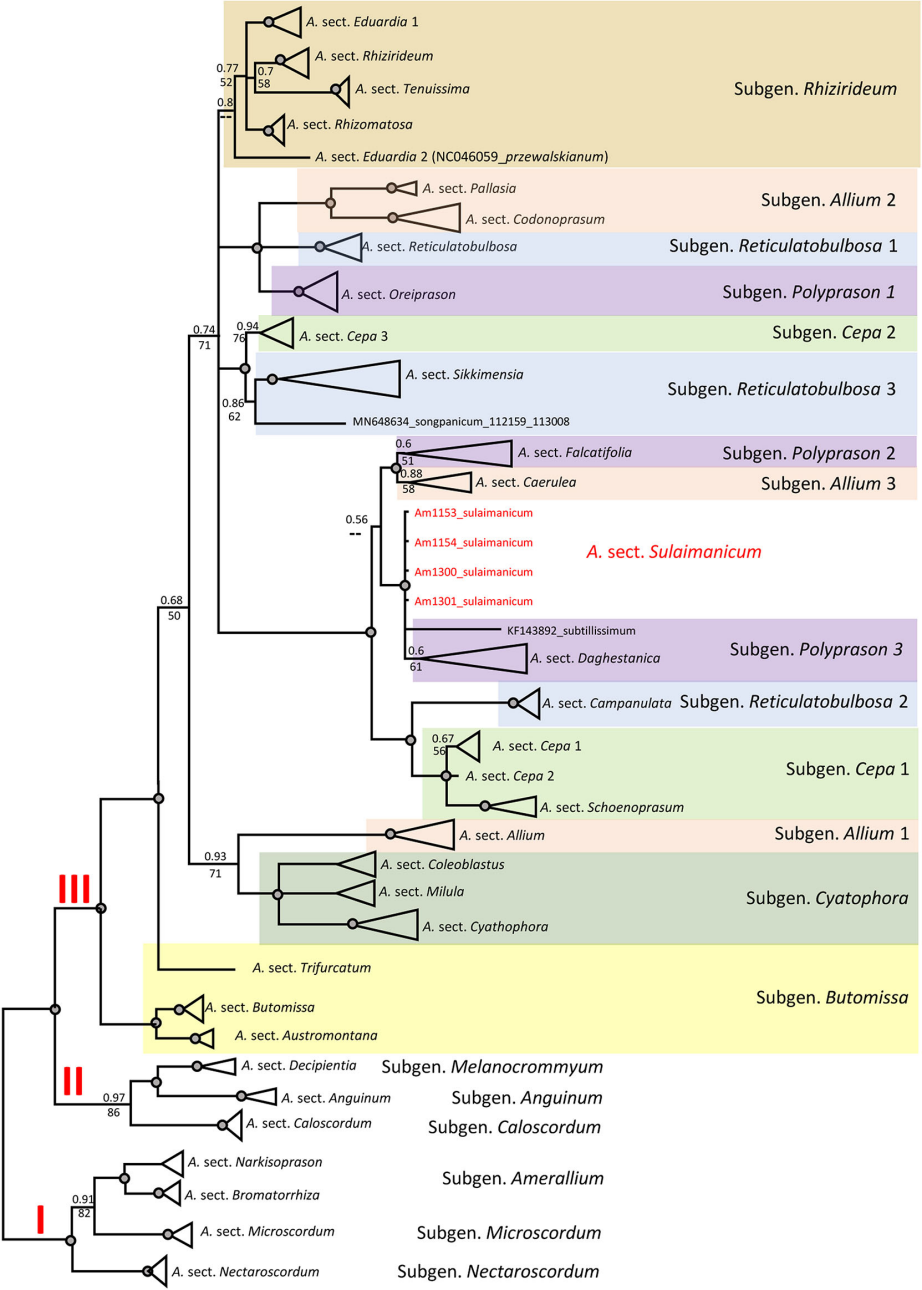
Generalised CP tree (*rpl*32-*trn*L (UAG) intergenic spacer) of the third evolutionary line of genus *Allium*. Numbers by nodes represent bootstrap support (1,000 replicates) and Bayesian probabilities. Roman numerals (I, II, and III) designate clades of three evolutionary lines. The joint presence of Bayesian probabilities over 0.98 and bootstrap support over 95% is indicated with a black dot.

The topology of the plastid tree, based only on an intergenic spacer (*rpl*32-*trn*L), is very different from the ITS tree. All four accessions of *A. sulaimanicum* were not resolved in the strongly supported clade (PP = 1; BS = 100) with *Allium* sect. *Daghestanica* and *Allium subtilissimum* Ledeb., whereas all species of *Allium* sect. *Daghestanica* formed a weakly supported clade (PP = 60; BS = 61) (see **Figure S2**). The generalised plastid tree with sections and subgeneric names is shown in **Figure 5**. The well-supported clade with representatives of the two *Allium* sections *Falcatifolia* and *Coerulea* form a sister clade to the clade with *A. sulaimanicum* but with very weak support (PP = 0.56 and no support in parsimony analysis). Furthermore, the *A. sulaimanicum* accessions belong to a strongly supported clade with representatives from the *A.* subgenera *Reticulatobulbosa* (only *Allium* sect. *Campanulata*) and *Cepa.* TrnQ-rps16 sequences are not known for all sections in the third evolutionary lineage and therefore could not be considered in the analysis.

### Cytological analysis

**Figure 6:**
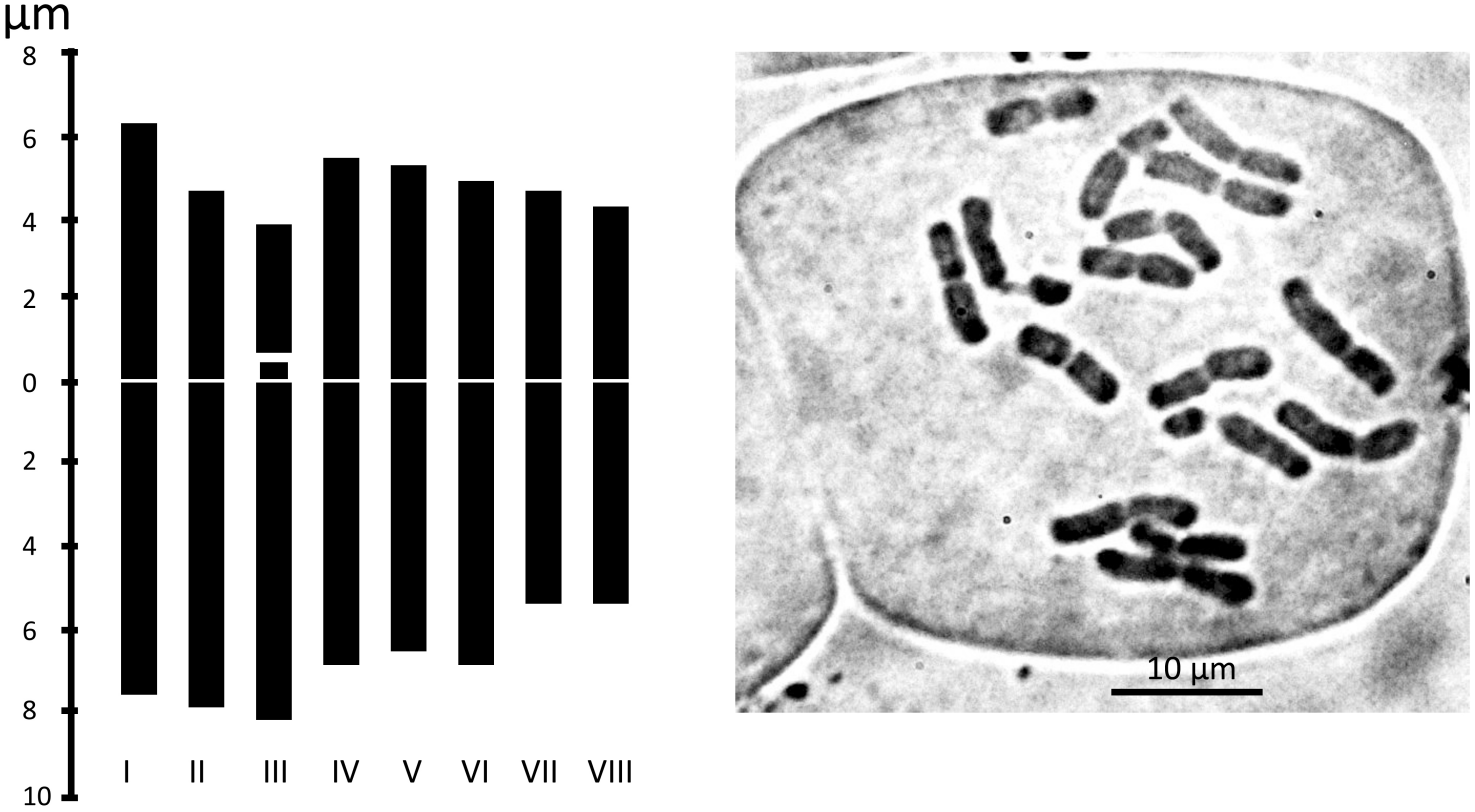
Metaphase plates and Idiograms of Allium sulaimanicum (Am1301). For the origin of the accession, see the **Table 1**.

We examined the chromosome number and karyotype only from accession Am1301. The plant is diploid with 2n = 16. There are 14 metacentric chromosomes and two telocentric chromosomes with a big satellite (**Table 2** and **Figure 6**).

**Table 2:** Karyo-morphometric parameters of *Allium sulaimanicum*.

Chromosome pairs	TAL (µm)	Long arm (µm)	Short arm (µm)	Satellite (µm)	CI %	Type
1	13.89	7.50 ± 0.91	6.39 ± 0.99		46.02	m
2	13.02	7.89 ± 0.73	5.14 ± 0.61		39.44	m
3	12.59	8.32 ± 0.54	0.64 ± 0.19	3.63 ± 0.19	5.10	t
4	12.48	6.84 ± 0.85	5.64 ± 0.57		45.22	m
5	11.49	6.25 ± 1.32	5.24 ± 0.84		45.62	m
6	10.89	6.08 ± 0.83	4.81 ± 0.78		44.16	m
7	9.89	5.36 ± 0.51	4.53 ± 0.71		45.78	m
8	9.04	5.36 ± 0.51	4.13 ± 0.62		45.69	m

Type chromosome nomenclature according to Levan et al. (1964). Total karyotype diploid length = 186.58 µm. Karyotype centromeric index = 39.1%.

TAL, total absolute length; CI, centromeric index.

## Discussion

Some subgenera in the third evolutionary lineage according to Friesen et al. (2006) are not monophyletic in both nuclear (ITS) and plastid (*rpl*32-*trn*L) analyses, but the differences are not identical. This concerns the *Allium* subgenera *Cepa*, *Reticulatobulbosa*, *Polyprason*, and *Allium*. These results agree with previously published phylogenetic analyses (Li et al., 2016; Hauenschild et al., 2017; Xie et al., 2019; Friesen et al., 2020a; Xie et al., 2020; Friesen, 2022). The phylogenetic consequences for the non-monophyletic subgenera should be drawn from a detailed analysis in the future. The position of *A. sulaimanicum* inside the third evolutionary lineage is the most significant finding of the current study. In the ITS tree, the accessions of *A. sulaimanicum* form a separate monophyletic clade inside the third evolutionary lineage with strong support: Bayesian PPs = 1 and BS = 100 (see **Figures 4**, **S1**). In the plastid trees (**Figures 5**, **S2**), accessions of *A. sulaimanicum* form a strongly supported clade together with species of *Allium* sect. *Daghestanica* and *A. subtilissimum*. The sister group here is the clade with representatives of the *Allium* sect. *Falcatifolia* and *Allium* sect. *Coerulea*, which correlates well with blast analysis.

These discrepancies between nuclear (ITS) and plastid (rpl32-trnL) trees clearly point to a possible hybridogenic origin of *A. sulaimanicum*. One of the progenitors of *A. sulaimanicum* was certainly a representative of the *Allium* sect. *Daghestanica*, but the second parental taxon cannot be concluded from these data. Here, only the sequencing of the full genome can bring clarity. It should be noted that species of *Allium* sect. *Daghestanica* are distributed in three isolated areas: Europe, Caucasus, and China (Xie et al., 2019; Friesen et al., 2020b; Li et al., 2021). *A. sulaimanicum* is located in the large gap between Caucasian and Chinese localities. Another evidence of the relationship to some representatives of *Allium* sect. *Daghestanica* is the similarity of satellite chromosomes in *A. sulaimanicum* with satellite chromosomes in *A. maowenense* J. M. Xu (Xu et al., 1994), which also has telocentric chromosomes with very long satellites. It should be mentioned that other species in the section *Daghestanica*, in which the chromosomes were studied, have satellite chromosomes with very small satellites (*A. chrysocephalum* Regel and *Allium herderianum* Regel, Hongguan et al., 2005; *A. gunibicum* Miscz. ex Grossh., Zakirova and Vakhtina, 1974; *Allium ericetorum* Thore, *Allium suaveolens* Jacq., and *Allium ochroleucum *Walldst. & Kit., Májovský and Murín, 1985; Wetschnig, 1992).

Morphology of inflorescence and flowers shows some similarity with representatives of *Allium* sect. *Daghestanica*, the outer coats of bulbs are also similar, and only the long runner-like rhizome occurs rarely in the genus *Allium* (e.g., in *Allium caespitosum* Siev. ex Bong. & C.A.Mey from *Allium* sect. *Rhizomatosa* (Friesen et al., 2020a) and in some species in the section *Allium* (Mathew, 1996). The colour of the flowers is similar only in *A. maowenense* and *A. matiniae* from *Allium* sect. *Daghestanica*. All other representatives of Chinese species from *Allium* sect. *Dagestanica* have yellowish colour (see photos of all Chinese species of *Allium* sect. *Daghestanica* in Xie et al., 2019; Li et al., 2021). *A. sulaimanicum* was rarely detected hitherto, and we would like to encourage the scientific community to look for this new species whose morphological variation and ecological preferences remain insufficiently known yet.

## Data availability statement

The data presented in the study are deposited in the NCBI GenBank, Accessions number OP218571 - OP218574, OP210288 - OP210295. Alignments are deposited in TataDryad doi: 10.5061/dryad.73n5tb313.

## Author contributions

NK: Data curation, Formal analysis, Writing - review and editing. NF: Conceptualization, Formal analysis, Data curation, Writing - original draft, Writing - review and editing. AS: Resources, Data curation, Writing - review and editing. RF: Data curation, Formal analysis, Writing - review and editing., TK: Writing - review and editing. KI: Writing - review and editing. All authors contributed to the article and approved the submitted version.
