# Supplementary material for: Allium sulaimanicum: A new Allium species and section from Pakistan
Source: Front Plant Sci. 2022 Dec 14;13:1020440. doi: 10.3389/fpls.2022.1020440 (PMC9795052; doi:10.3389/fpls.2022.1020440)
Supplement: Supplementary file 1 [file DataSheet_1.docx]

**Supplements**

**
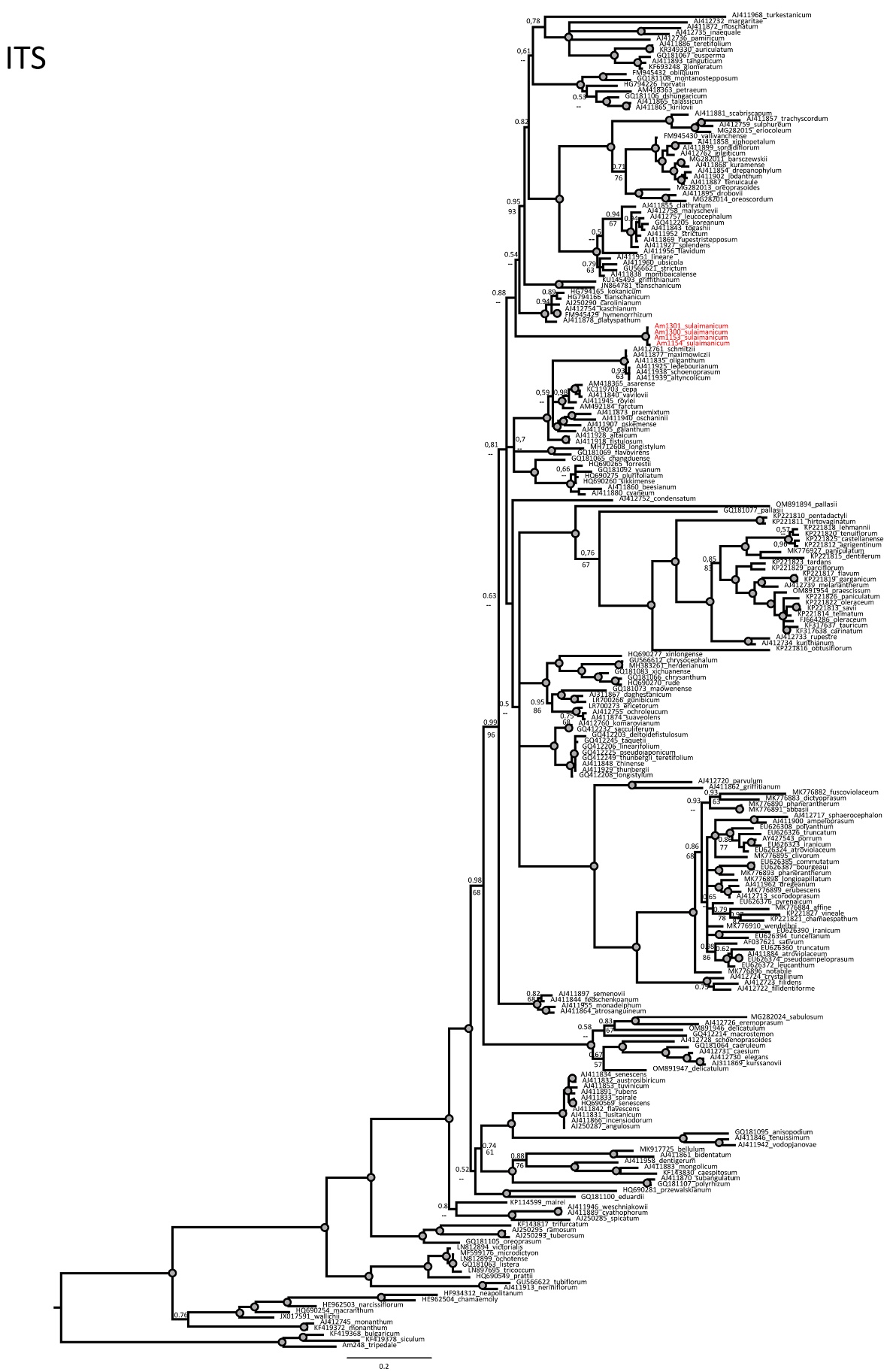
**

**Figure S1.**  Phylogenetic tree of third evolutionary lineage of the genus *Allium*, based on ITS sequences from NCBI GenBank. Numbers by nodes represent bootstrap support (1000 replicates) and Bayesian probabilities. The joint presence of Bayesian probabilities over 0.98 and bootstrap support over 95% is indicated with a black dot. Origin of samples without GenBank accession number see in Table 1.

**
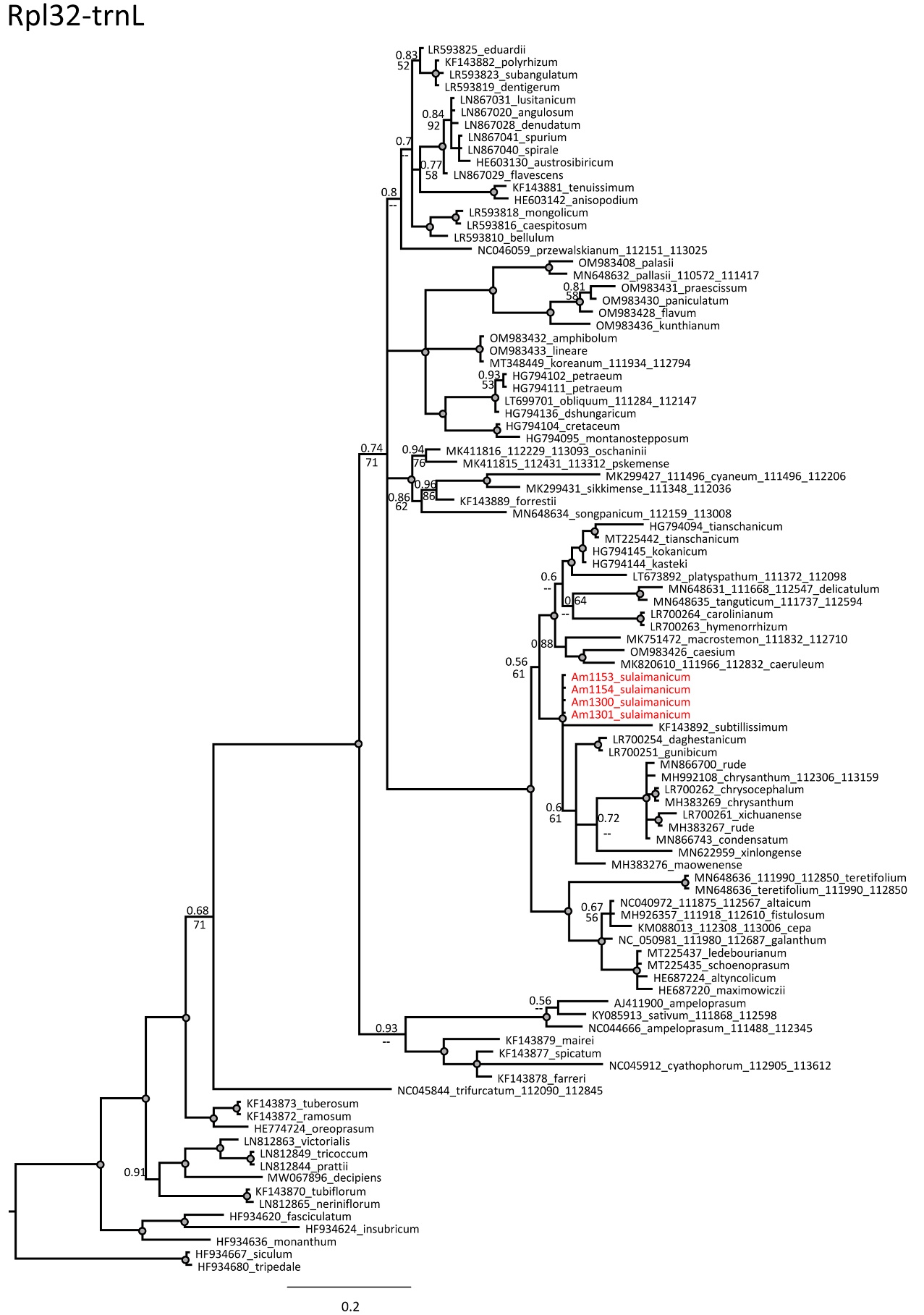
**

**Figure S2.** Phylogenetic tree of third evolutionary lineage of the genus *Allium*, based on CP DNA sequences (*trn*L-*rpl*32) from NCBI GenBank. Numbers by nodes represent bootstrap support (1000 replicates) and Bayesian probabilities. The joint presence of Bayesian probabilities over 0.98 and bootstrap support over 95% is indicated with a black dot. Origin of samples without GenBank accession number see in Table 1.
